# The impact of and responses to flooding in Thulamela Municipality, Limpopo Province, South Africa

**DOI:** 10.4102/jamba.v8i2.166

**Published:** 2016-01-13

**Authors:** Agnes Musyoki, Reuben Thifhulufhelwi, Florence M. Murungweni

**Affiliations:** 1 Department of Geography & Geo-Information Sciences, University of Venda, South Africa; 2 Department of Environmental affairs, Pretoria, South Africa; 3 Department of Ecology & Resource Management, University of Venda, South Africa

## Abstract

The frequency of climate-related disasters such as floods is growing due to environmental and human factors. This paper examines the impact of flooding and communities’ perceptions towards responses to flooding in the cases of Maniini and Tshilungwi Villages in the Thulamela Municipality in the Limpopo Province of South Africa. A questionnaire survey was conducted with 60 household respondents in the two selected villages and then analysed. Key-informant interviews with community leaders and municipality officials established the key impact of and responses to the flooding. Secondary data on flooding provided useful historical trends in the region. Field observations assisted in corroborating information provided in interviews. The findings indicate that communities are vulnerable to flood disasters, and these disasters had a significant impact on infrastructure and the livelihood of the selected communities. An increase in household income and levels of education as well as access to grants decreased households’ vulnerability in cases of flooding. The responses to flooding by the municipality were viewed negatively by the community who did not support permanent relocation. Hence, the article points out the need to strengthen coping mechanisms by local governments and communities themselves in order to cope with the impact of flooding.

## Introduction

Flood disasters are growing in frequency worldwide due to a variety of environmental and human factors. Natural disasters and floods in particular are becoming more frequent and destructive (Garcia-Castellanos *et al.* 2009; Li *et al.* 2014; Miller 1997; Smith 2001).

Physical causes of floods include the nature of precipitation, topography, vegetation, soil type and runoff patterns whilst human factors contributing to flooding are mostly associated with development and land use (Khan 2004; Smith 2001). Over the last 50, years anthropogenic factors have played a determining role in causing flood disasters (Clements 2009). The impact of flooding includes destruction of and damage to roads, bridges, buildings or sewage-disposal systems. Flood disasters affect agricultural production, bringing about food insecurity in communities (FAO–SAFR 2002).

Governments in southern Africa are devising mechanisms to cope with the impact of flooding, but these efforts are often limited and are affected by people’s perceptions which in turn influence their responses. Communities often resist relocation as flooding is an essential component of agriculture, and the ecological systems provide the basis for the regeneration of crops, plants and aquatic life (Wisner *et al.* 2004). A report by the Limpopo Department of Agriculture (2002) indicates that apartheid created conditions that were unfavourable to the welfare of rural Black people. This historical legacy led to the present situation where the Black majority stay in poor marginal land that is inadequate for subsistence farming and prone to flooding.

**FIGURE 1:**
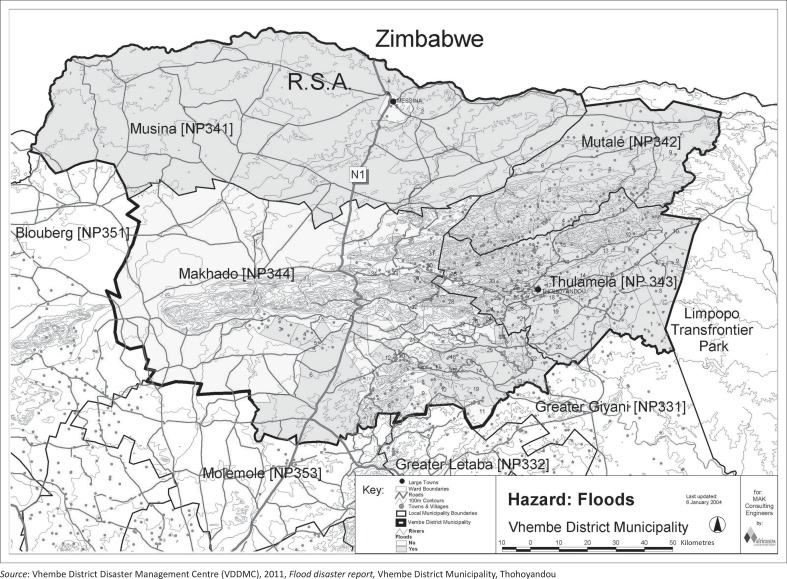
Location of Vhembe District Municipality in South Africa and all areas vulnerable to flood hazard. Dark grey areas are more vulnerable to flooding (Musina, Mutale and Thulamela).

Thulamela municipality is vulnerable to hazards such as diseases, fires, drought, thunderstorms and flooding. This is associated with high levels of poverty, landlessness and poor infrastructure. The physiography of the area and the location of settlements render Thulamela communities prone to flooding. Before and after the 1999–2000 flood, disaster management for floods was found to be lacking and ineffective. This was the first major flood disaster, caused by tropical cyclone Eline, but the municipality was completely unprepared according to Mudinda (2010). Ten years later, flooding became a major disaster once again. This last flood (2010–2011) led to this study in the two villages of Maniini and Tshilungwi in the Limpopo Province. Figure 1 indicates the location of the Vhembe District Municipality and areas that are vulnerable to floods.

Research on the impacts of and responses to flooding have been undertaken at national, provincial and city level and focused on the causes and impact of floods (Mudau 2001; Sengani 2008). Few studies have been done at local level in the rural areas of South Africa. There are no studies on the impact of flood disasters in these two villages (Maniini and Tshilungwi), this despite the high level of vulnerability.

### Statement of problem

During the rainy season of December 2010 and January 2011, flooding resulted in enormous destruction of infrastructure and property. Limpopo Province was one of the heavily affected provinces with 1540 houses, private properties and schools damaged as reported by the provincial government. In the Vhembe District Municipality, 632 houses were damaged, and in the Thulamela Local Municipality, 246 houses were damaged (Vhembe District Disaster Management Centre 2011).

In January and February 2011, the flooding disaster raised questions on the flood vulnerability and disaster-management strategies employed by Thulamela Municipality. The community’s perceptions, experiences and responses were neither clearly understood nor were these incorporated into plans and programmes for disaster management in the municipality. Hence, this exploratory research aims to examine the impact of floods and community perceptions and experiences in order to recommend new strategies to minimise the impact of flooding in Thulamela Municipality.

### Specific objectives

The aims of this article is to:

- identify and describe the impact of the 2011 flood and the experiences in the Tshilungwi and Maniini communities in Thulamela
- examine community perceptions concerning the response to the flood and coping strategies in Thulamela.

### Literature review

A flood is an excessive water flow into normal dry lands. It is an inundation of normally dry areas caused by rising water in an existing waterway such as river, stream or drainage ditch (Clements 2009). Notwithstanding the fact that water is a necessity of life because all living things depend on it, water at the wrong time or place can expose the community to danger. Excessive amounts of water or its intrusion into areas reserved for other purposes represents a hazard to people’s lives, and it also becomes the most frequent and demanding of all types of natural disasters (Smith 2001).

Flash floods are floods of short duration with a relatively high peak discharge and are often caused by heavy or excessive rainfall over a short period of time. Flash floods are distinguished from a regular fluvial flood by a timescale of less than six hours (Xia *et al.* 2011). Flash floods often result from high-intensity precipitation such as thunderstorms. This type of flooding usually occurs in mountainous areas where steep slopes favour the rapid flow of water and in arid areas where the poor absorptive capacity of arid soil leads to excessive runoff. Flash flooding often results in a catastrophic impact and present a potential threat to people’s lives and livelihood because of the limited opportunity for issuing warnings and undertaking some precautionary measures before the disaster.

Human-induced climate change and climate-related disasters are expected to wreak havoc on people and their property in the affected community (Canesio 2010). The Intergovernmental Panel on Climate Change (IPCC) (2007) also predicts that heavy precipitation is very likely to increase in frequency, and the resultant floods will affect life and livelihoods. Naess *et al.* (2005) argue that the nature and extent of the impact of climate-related disasters depend on the type and intensity of the disaster and on the vulnerability and preparedness of people in the affected community. However, Wisner *et al.* (2004) argues that, in coping with climate-change effects, there is a need for monetary resources and a range of expectations to achieve various defensive mechanisms and methods of handling stress.

#### Flood forecasting, preparedness and vulnerability

Flood forecasting and early-warning systems increase the awareness of and preparedness for a potential disaster. Von Kotze and Holloway (1996) describe preparedness for a disaster as measures that ensure the readiness and ability of a society to forecast and take precautionary measures before a disaster and to respond to or cope with the effects of a disaster by organising and delivering timely and effective rescue, relief and other post-disaster assistance. Conducting campaigns to educate household, monitoring rainfall and reporting through radio, television and other form of media also form part of preparedness for a disaster (World Bank 2006).

Von Kotze and Holloway (1996) defines vulnerability as the extent to which an individual, community, structure, service or geographic area is likely to be damaged or disrupted by the impact of a particular disaster. Furthermore, Lewis (1999) argues that natural disasters occur only where the extremes of the environment meet a human community that is vulnerable, and the conditions of vulnerability comprises numerous inter-related components and factors such as location, age, socio-economic conditions, access to resources and services and political integration. In the context of floods, vulnerability is referred to as the measure of risk combined with the level of social and economic ability to cope with a flood event (Smith 1996).

#### Coping strategies

Wisner *et al.* (2004) argue that almost all effective coping strategies for adverse events such as floods consist of actions before, during and after the event. The major coping strategies include preventive, impact-minimising or mitigating and post-event coping strategies. Preventive strategies attempt to avoid the disaster from happening in the first place. They involve active public participation and strong political mobilisation to ensure implementation at large scale. Preventive strategies include the construction of dykes, flood banks or levees to divert the flood water, the building of dams and reservoirs to retain flood waters upstream and the improvement of canals to evacuate flood waters more rapidly (Smith 2001).

Non-structural flood-defence measures such as land-use planning and zoning, flood proofing of existing structures and flood forecasting and preparedness are often implemented. The main aim is to keep people out of the way of the flood, and these measures can be very effective when implemented together with structural measures (Dawson *et al.* 2011).

#### Flood-plain management and disaster management

A flood plain is the land subjected to flooding next to a river channel, and it is on this zone that flood damage is likely to occur. This can be done by preventing the inundation of flood plains or alternatively by controlling the development on a flood plain so that flood damage is reduced. This alternative has become more popular in recent decades, particularly on environmental grounds, but may not always be practical. Flood plains attract human settlements and development since they are generally flat, located near water and usually fertile. If there are no alternative areas available, development will continue on the flood plain (Viviroli *et al.* 2007).

It is important to reduce the impact of human-induced flood disasters, which are often associated with farming and settlement in flood zones. Flood maps can be prepared, varying from simple maps of areas flooded in the past to comprehensive maps showing the areas that would be flooded and the probability of such an event. However, the most successful form of flood-plain map which shows the areas that will be flooded, and its probability, requires an accurate survey of the river channel and the flood plain (Pelling 2003).

Disaster management encompasses risk assessment, a vulnerability analysis, preparedness, prevention and measures for mitigation, recovery and rehabilitation (Von Kotze & Holloway 1996). These processes work like a system, and all components must be integrated to ensure effective disaster management. The isolation of any one component can affect the whole process.

The ‘contract or expand disaster model’ assumes that all components of disaster management can be carried out at all times in a hazard-prone community. The weighting of each component expands or contacts, depending on the vulnerability of the community (Von Kotze & Holloway 1996). The model also assumes that disasters occur when a hazard exceeds a community’s capacity to cope. If this takes place, then relief, response, rehabilitation and emergency evacuation must be undertaken. Previous studies on flooding in the Thulamela Municipality have indicated poor management by the municipality and a lack of preparedness by communities who are often faced with disaster situations which could have been avoided (Mudau 2001; Sengani 2008). The adequate management of floods must, however, be based on solid evidence from the affected areas. Hence, we explore the impact of flooding and community perceptions on responses by Thulamela Municipality officials and community members in the two villages of Maniini and Tshilungwi in the Limpopo Province of South Africa.

## Research setting

**FIGURE 2:**
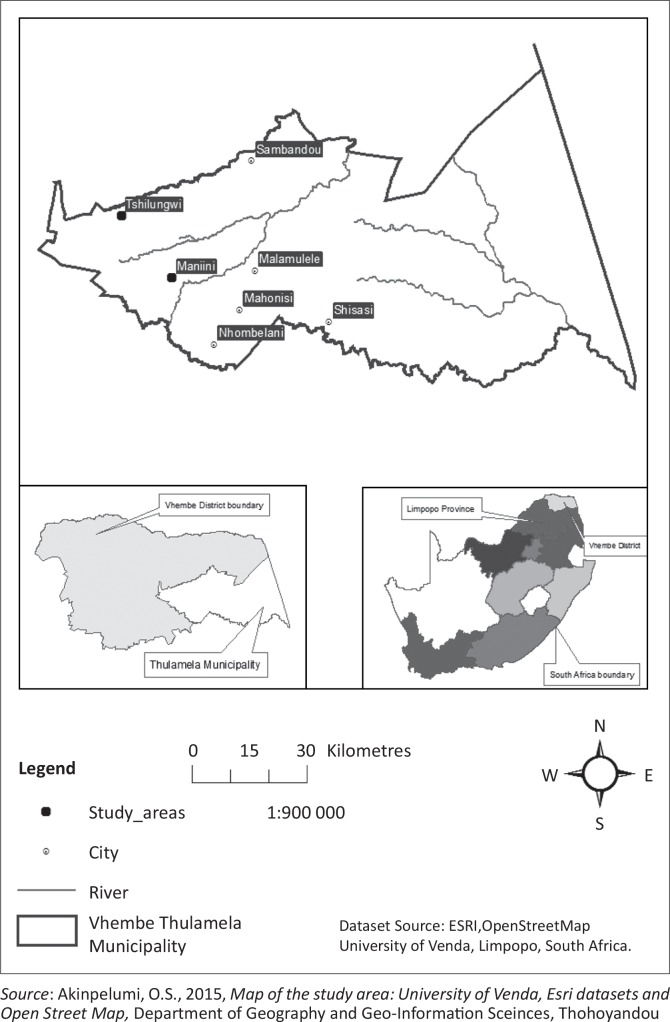
Map of study area.

Thulamela Municipality is one of the four local municipalities in the Vhembe District of Limpopo (Figure 2). The major town in this municipality is Thohoyandou. Thulamela’s population is 618 462, 54.4% of whom are women (Statistics South Africa/Census 2011). The municipality is situated in the eastern subtropical region of the province, and it is generally hot and humid. It receives much of its rainfall during summer, from November to March, as the inter-tropical convergence zone (ITCZ) moves south (Kabanda 2004). The Maniini and Tshilungwi study sites are characterised by a dry season in the months of March to September. The average temperature ranges from 35 °C in summer to 18 °C in winter (Nethengwe 2007). The geology and soil of the sites consist of fertile clay soil, sandy soil and clay loam soil. The land use of the municipality includes residential, subsistence agriculture and commercial uses in Thohoyandou town (Figure 2). Informal businesses within and around the town plays a significant role in the people’s economic and social life.

We purposively sampled these villages because of experiences with flooding and community reports of the negative impact of the flood disaster of January–February 2011.

Most research on the impact of flooding is done at national and city levels, and few studies are done at the local level in rural areas in South Africa.

### Tshilungwi Village

Tshilungwi has a population of 663 people, constituting 0.12% of Thulamela’s population (Statistics South Africa/Census 2011). The village’s terrain is mostly gently rolling plains, mountainous and hilly. Agriculture is the dominant land use.

### Maniini Village

Maniini Village is within Thohoyandou town, occupying blocks K, L and M as a result of new demarcation of town boundaries. It has a population of 13 644, representing 2.2% of Thulamela’s population. Maniini has gentle terrain and is located in a flood, plain making it vulnerable to flooding.

### Material and method

We used a case-study approach in two villages in Thulamela which allowed the investigation of a particular event of flooding. We selected these two villages in order to show the impact of and the response to flooding at a local level. The two villages display differences in physiography with one a low-lying area near river and the other an undulating plain. A mixed-method approach using both qualitative and quantitative survey designs was adopted for the purpose of data collection and analysis.

### Sampling

We obtained a list of affected residents, and using systematic random sampling, 35 households from Maniini and 25 households from Tshilungwi were selected and interviewed. We conducted key-informant interviews with two officials from Vhembe District’s Disaster Management Centre (VDDMC), with one official from Thulamela Municipality, with one community leader from each of Maniini and Tshilungwi and with the chief of Tshilungwi Village. We selected these two villages based on reports by the community members and on information from the Municipality which identified them as some of the most vulnerable in the district.

### Secondary data

The main source of secondary data was the Census 2011 document which provided us with information regarding total village population, housing types, infrastructure and population density. All photographs including those of collapsed houses, damaged roads and bridges were obtained from Vhembe District Disaster Management Centre (DDMC) records.

### Primary data

#### Household survey

We conducted the household survey at Maniini and Tshilungwi over a period of three months between June and August 2012. We distributed questionnaires to literate households and collected them for analysis. We assisted all the illiterate households by translating and explaining the questions in Tshivhenda (their mother tongue) and recorded the responses. Our analysis involved labelling and coding data using Statistical Package for Social Sciences (SPSS) and cross tabulations of descriptive statistics. We then produced graphs and tables for interpretation. We were able to obtain information on socio-economic characteristics, including poverty level, literacy and per capita income. We used narratives from the open-ended questions to record and analyse people’s experiences and perceptions of the flood disaster. The responses provided us with information on the impact on services and infrastructure in order to understand the extent and severity of the flood event and hence the vulnerability of these communities to future events. We also carried out field observations to confirm information on the environmental and physical impact.

#### Key-informant interviews

In September 2011, we carried out in-depth interviews with two senior officials from VDDMC, one official from Thulamela municipality, two community leaders from Maniini and Tshilungwi and the chief of Tshilungwi Village. We made two visits each to the municipal offices and the villages where, using an interview guide, questions were posed and answers recorded in narrative form. These were later analysed and themes identified, collated and discussed. We were able to obtain information on experiences during the flood, coping mechanisms, causes of flooding and its impact on agriculture, infrastructure, housing and education. Key informants assisted us in weighting the responses.

## Findings, interpretation and discussion

### Causes of flooding

The results show that flooding in Thulamela municipality was caused by torrential rain which affected most of southern Africa in 2010–2011. This led to tremendous damage and significant impact on households and community members. Amongst all four local municipalities within the Vhembe District, Thulamela municipality was hardest hit with approximately 246 damaged houses, roads and other non-quantified damage estimated to cost around 500 million South African Rand (ZAR). Torrential rainfall due to several thunderstorms caused flooding in most parts of Thulamela Municipality. The household survey showed that human activities such as cultivation on steep slopes (16%) and the clearing of vegetation (12%) had contributed to in floods, especially at Tshilungwi Village. Excessive heavy rainfall over a short period of time caused the flood, affecting 48% of all respondents in Tshilungwi and 66% in Maniini Village (Table 1). The community was very knowledgeable on the causes of floods (Table 1).

**TABLE 1:** Community perceptions on causes of the flood disaster in Tshilungwi and Maniini Villages.

Major causes of flooding as reported by survey respondents	Tshilungwi Village (*N* = 25)		Maniini Village (*N* = 35)	
	
	Number of responses (*f*)	% of cases	Number of responses (*f*)	% of cases
Excessive heavy rainfall	12	48	23	66
Cultivation on steep slope	4	16	0	0
Vegetation clearing	3	12	0	0
Effect of relief	4	16	5	14
Inhabitation of flood plains	2	8	7	20

**Total**	**25**	**100**	**35**	**100**

The effect of relief was also important as indicated by 16% of respondents from Tshilungwi and 14% (Table 1) from Maniini Village. Relief played a major role in both study areas because both villages are situated at the foot of the mountain, with Maniini Village at a very gently slope and Tshilungwi situated between mountains. Therefore, at Tshilungwi Village, rugged mountains increased the runoff rate and the erosive power of water which resulted in flooding down the mountain. At Maniini Village, the slope is very gentle, and the area is located next to the river. Therefore, during heavy rain seasons, as occurred in December 2010 – January 2011, the river overflowed its banks, which resulted in flooding of houses close to the flood line.

### The impact of flooding and the experiences of communities

Data from both villages show that the biggest impact was on the roads. This was confirmed by 60% of all respondents in Tshilungwi Village and 57% in Maniini Village (Figure 2). The second biggest impact was the destruction of agricultural land and livestock. This resulted in significant impact on households’ welfare, their socio-economic conditions and the livelihood systems of the communities as noted by one community member:

This flood has caused us a lot of problems—we lost our grazing lands and livestock. The flooding is getting worse as time goes by. We never had such flooding before 1999. (Lady, subsistence farmer, 46)

### Damage to infrastructure

**FIGURE 3:**
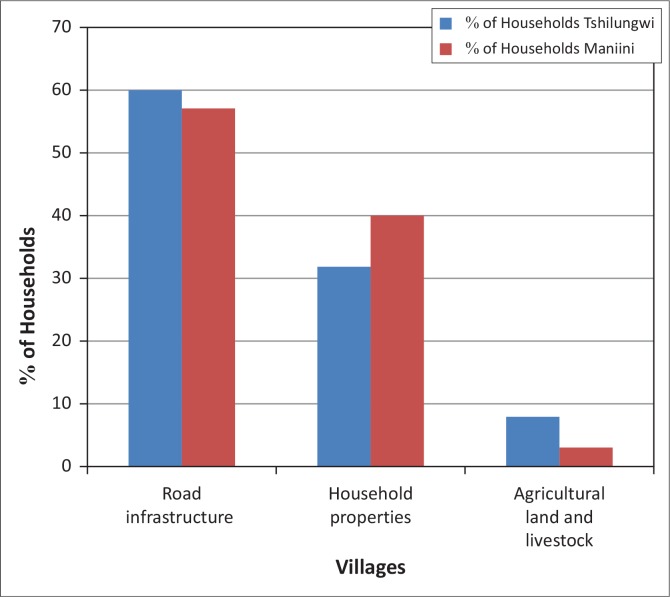
Flood damage at Tshilungwi and Maniini Villages, December 2010 and January 2011.

**FIGURE 4:**
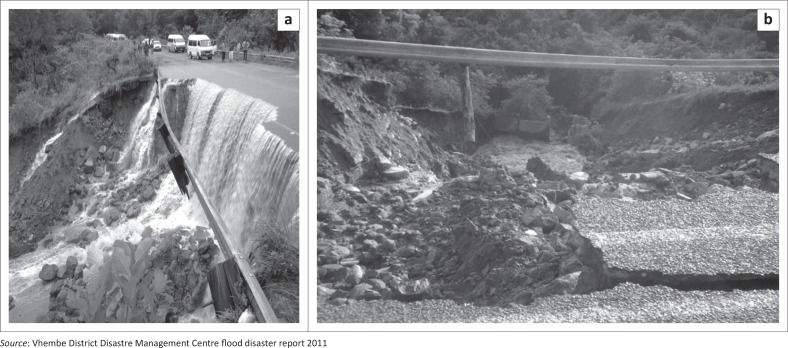
Total collapse of Nzhelele to Sibasa bridge (18–21 January).

According to reports from the VDDMC, observations in the field and interviews, the 2010–2011 flood disaster in Thulamela Municipality caused tremendous damage to roads, telephone lines and other public infrastructure (Figures 3 and 4).

The damage to the roads hampered access to the two major towns within the municipality (Thohoyandou and Nzhelele), and more than 100 villages and communities were affected in terms of access to the work place, school, shopping and health facilities. The recovery or rehabilitation measures of constructing a fully functional bridge was estimated to cost approximately R162 million.

**FIGURE 5:**
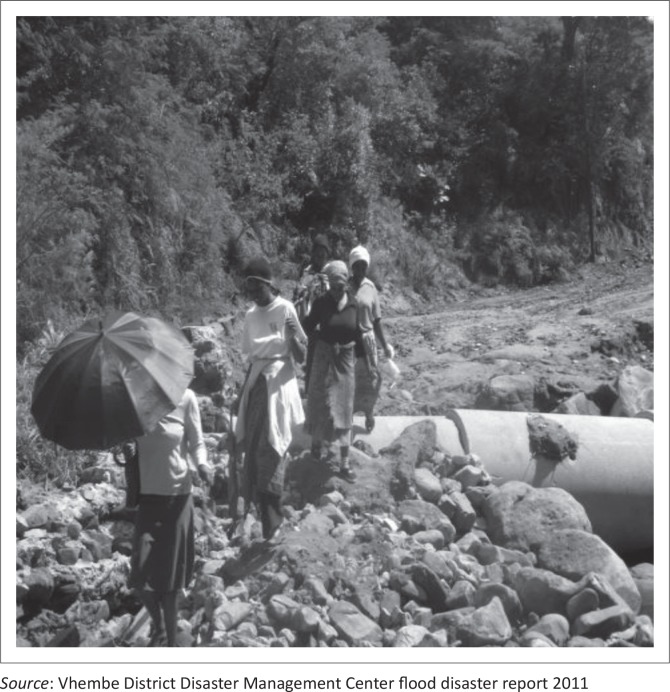
Damage to road and culvert (Road D3688) – Tshilungwi Village.

Furthermore, Figure 5 also shows how the Tshilungwi road was damaged. Large boulders of rocks were washed onto the road. Bad drainage around the bridge contributed to it being washed away.

The construction of a bigger culvert structure and upgrading the road were estimated to cost approximately R110 million.

### Damage to houses and schools

**FIGURE 6:**
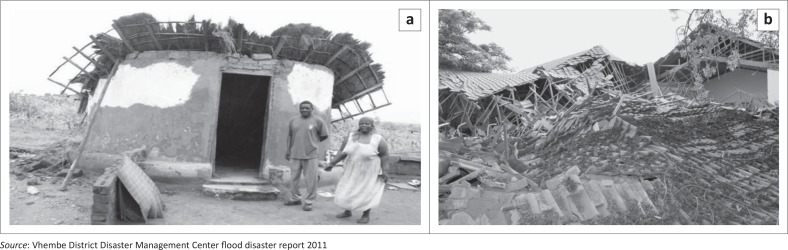
Houses crumbled by flood at (a) Tshilungwi and (b) Maniini.

Approximately 246 houses, including schools, were damaged. Learning programs were affected as 23 classrooms at Maniini Primary School were damaged by a thunderstorm. Four mobile classes were supplied as immediate relief (Figure 6) with some victims accommodated by their relatives and friends.

The examples above clearly show that flooding is a serious problem for the Tshilungwi and Maniini communities. They have been disrupted by this particular disaster that affected transportation, access to food resources, schools and housing. These communities show high levels of vulnerability due to the geography and the socio-economic status of their inhabitants. It is interesting to note that community members are very much aware of both anthropogenic and natural causes and the impact of flooding.

## Responses by the municipality, government, civic organisation and other local institutions

Various local-government units played a vital role in the recovery and rehabilitation measures as part of the response after the disaster had taken place. Food parcels, tents and other necessary commodities were supplied to the affected communities.

Local businesses also contributed to disaster-relief funds. However, all the effort made by the Municipality and other local-government units was considered to be temporary and short-term. This is because they mainly focused on the recovery and rehabilitation measures with little focus on disaster preparedness, mitigation and risk management. During the household survey, participants were asked about the role of government units before, during and after flood disasters. In response, 80% of Tshilungwi and 66% of Maniini respondents indicated that local-government units were actively involved only after the disaster. This is because the municipality lacked the capacity for the implementation of long-term strategic planning. Preparedness and preventive measures before the disaster were not adequately implemented as indicated by officials.

## Socio-economic status, flood vulnerability and coping strategies

We utilised a household survey in examining the socio-economic status and coping mechanisms utilised to mitigate the impact of the flood. Particular attention was paid to socio-economic characteristics such as the age of respondents, their level of education, their type of house and the income and household size because these are important indicators for the assessment of a household’s vulnerability to a flood.

**FIGURE 7:**
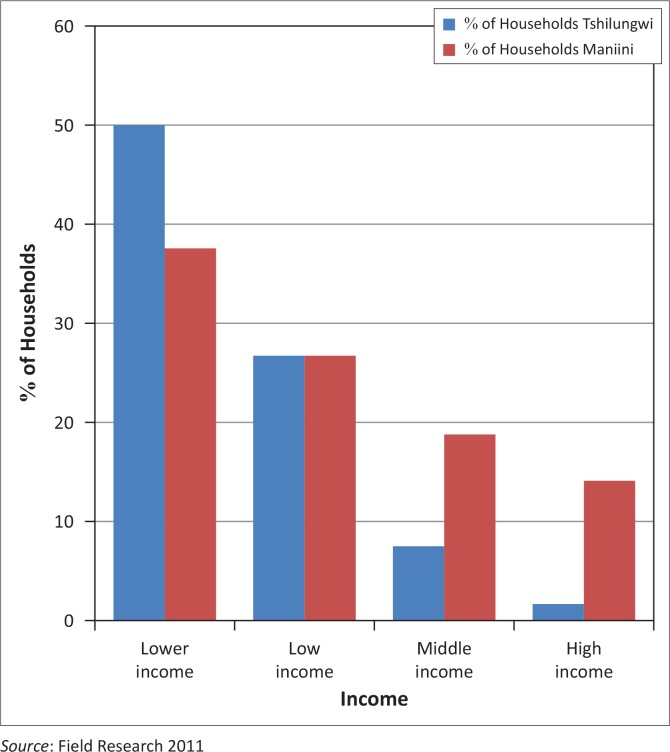
Annual income profile of households in Maniini and Tshilungwi.

Survey results showed that households with a low annual income were the most vulnerable and affected more by floods (Figure 7). This is because coping with a flood disaster demands financial reserves that can buffer the household against the negative impact. Furthermore, the findings also indicate that households with higher levels of education had higher annual income and thus coped better than low-income households. This indicates the importance of education in vulnerability assessment.

Household incomes are divided into four different groups from the poorest, lower income to the highest as indicated in Figure 7. The specific categories for income per month are as follows: lower income (R500 and below), low income (R5001 – R10 000), middle income (R10 001 – R20 000) and high income (R20 001+). Results show that the majority of respondents from both villages (Tshilungwi 52% and Maniini 35%) fall in the lower-income category whilst only 4% of respondents in Tshilungwi and 16% in Maniini fall within the high-income category. One of the respondents in the low-income category noted:

Before the flooding we were able to go to work and carry out our normal life with the floods we were not able to go anywhere and our children and ourselves suffered. (Man, subsistence farmer, 56)

The reason for increased vulnerability might have been the respondents’ level of education as the majority of respondents (Tshilungwi 60% and Maniini 63%) had only a secondary education whilst 8% and 17% respectively had tertiary education. Respondents with tertiary education have a better chance of obtaining a good jobs that pays well, consequently reducing their vulnerability.

## Community perceptions and disaster-management strategies

We asked the heads of households to identify the adaptation strategies employed during the flood and how the local-government units addressed the impact of the disaster. We also asked households to choose one key adaptation strategy that they would use in a future flood. Finally, we assessed the effectiveness of all strategies and ranked them from the most preferred to the least preferred (Table 2).

**TABLE 2:** Multiple-response ranking of households’ strategies of coping with the floods in Maniini and Tshilungwi Villages.

Coping strategies for future disaster	Maniini Village†		Tshilungwi Village†	
	
	Number of responses (*f*)	Rank	Number of responses (*f*)	Rank
Covering, protecting /restructuring housing unit	18	1	5	4
Levees and terraces	13	2	13	2
Evacuation to place of safety	12	3	15	1
Disaster aid /insurance	9	4	5	4
Ask help from neighbours and friends	9	4	13	2
Ask for help from local businesses and government	7	5	10	3
Borrow money from friends, neighbours and relatives, but not banks	4	6	10	3
Do nothing /not applicable	2	7	3	5
Other	-	8	-	6

Scale 1 (most preferred) to scale 8 (least preferred).

† *N* = 25 Cases.

Strategies identified for coping with future disasters were ranked using a multiple-response ranking of the strategies that households used to cope with the flood, from the most preferred to the least preferred (Table 2). Survey results from both villages indicate that most people are prepared to take precautionary measures against future threats from climate-related disasters. Their strategies are summarised in Table 2.

In Maniini Village, covering or protecting a housing unit to avoid destruction whilst not moving from their residence was the most preferred alternative. In order not to have to move, community members built levees and terraces, and only if this was not adequate, they would be willing to move to a place of safety. Only when these two efforts fail would they seek help from insurance or disaster aid. It is surprising that asking help from government, borrowing money or doing nothing ranked low in their adaptive strategies.

At Tshilungwi Village, evacuation to a place of safety was considered the first priority, indicating a higher vulnerability than in Maniini. Only if evacuation was not possible or delayed would they seek to protect their houses by building levees and terraces and seek help from neighbours, friends or family and borrow money. Seeking disaster insurance and restructuring or covering the houses did not seem feasible in this village.

In both cases, community members have a negative perception of local-government assistance as a way of coping with floods. A Tshilungwi resident believes that flooding is getting worse from year to year:

In the past my family used to stay on the hilly side and during that time we did not face much food problems … We moved here to have access to water for our livestock … We are not prepared to relocate; we would rather evacuate during disaster times and come back. (Man, livestock farmer, 45)

## Discussion and conclusion

The vulnerability of communities to climate-related disasters is increasing, especially in the case of poor, rural communities living at the foot of mountains and in flood plains. Mechanisms to cope with flood disasters vary in scope and magnitude, depending on the impact of events and on the vulnerability of the community and households to such disasters (Canesio 2010). Our study, like that conducted by Mudinda (2010) on the flood disaster of 1999–2000, shows that Thulamela remains highly vulnerable to flooding, and hence, there is still need for improved and well-coordinated disaster risk management and improved risk-reduction strategies.

Households and communities in Thulamela were tremendously vulnerable during the flood disaster, and most damage was to public infrastructure. Apart from the physical impact, the flood disaster also had a significant impact on the households’ welfare, their socio-economic conditions and the livelihood systems of the communities. These findings confirm the findings of other studies in southern Africa where changing climatic conditions are a serious threat to communities (Motsholapheko, Kgathi & Vanderpost 2011).

The perceptions of households that had experienced flooding indicated a high vulnerability to the risk and threat of flooding, and their socio-economic status influenced their level of vulnerability. The unemployed, with low levels of education and low annual income, also tended to be more vulnerable. Our findings are similar to what Canesio (2010) found in Ormoc and Cabalian Bay in the Philippines concerning the experiences of households and communities. Rehabilitation and disaster-relief operations were the most common activities undertaken by municipalities immediately after the disaster. As such, disaster management by the municipality is limited to reacting after the disaster, and few early-warning systems were implemented. The most preferred option, namely temporary relocation or evacuation, might have been influenced by a lack of awareness programs that provide early-warning systems, hence people were neither aware nor prepared for the flood disaster. Cooperative mechanisms by communities are necessary to combat the impact of floods as most coping strategies were implemented by households. This is contrary to Wisner *et al.* (2004) who notes that effective strategies for preparedness and coping are required before, during and after the event.

Whilst predictions concerning climate change and its effects are made at a large spatial scale, there is a need to view their ramifications at local levels. Hence, this study has contributed to our understanding of the disastrous impact of floods and community perceptions at the local, rural-village level, taking into consideration the views of the communities and the responses by the local municipality. The study recommends the need for vulnerability and risk analyses, a focus on risk reduction, the identification of long-term adaptation strategies, early-warning systems and the introduction of disaster risk studies at all levels of education.
